# Exploring gender differences in the impact of sleep and fatigue on disease severity in rheumatoid arthritis: a moderated mediation model

**DOI:** 10.55730/1300-0144.6113

**Published:** 2025-12-01

**Authors:** Musa SALMANOĞLU, Habip YILMAZ

**Affiliations:** 1 Department of Internal Medicine, Sultan 2. Abdülhamid Han Training and Research Hospital, Health Sciences University, İstanbul, Turkiye; 2 Department of Anesthesiology and Reanimation, Sultan 2. Abdülhamid Han Training and Research Hospital, Health Sciences University, İstanbul, Turkiye

**Keywords:** Rheumatoid arthritis, sleep quality, fatigue, disease severity, gender differences

## Abstract

**Background/aim:**

Rheumatoid arthritis (RA) is often accompanied by fatigue and sleep disturbances, which aggravate disease severity. Gender differences in these interrelationships remain insufficiently understood. This study aimed to examine whether fatigue mediates the association between sleep quality and disease severity in RA, and whether these pathways differ by gender.

**Materials and methods:**

A single-center study was conducted with 68 RA patients (55.9% female). Disease severity was assessed using the Routine Assessment of Patient Index Data-3, fatigue using the Bristol Rheumatology Fatigue Multidimensional Questionnaire, and sleep quality using the Pittsburgh Sleep Quality Index (PSQI).

**Results:**

Fatigue significantly mediated the relationship between poor sleep quality and disease severity (indirect effect β = 0.209, p = 0.003). While gender significantly predicted fatigue (β = 0.297, p = 0.005) and females reported higher fatigue and disease severity, gender did not significantly moderate the mediation pathway (PSQI × gender interaction β = 0.019, p = 0.856). The direct effect of sleep quality on disease severity was not significant (β = 0.047, p = 0.663), supporting a full mediation model. Menopausal status was not significantly related to symptom variation among women with RA.

**Conclusion:**

Fatigue is a key mechanism connecting poor sleep to greater disease severity in RA. The female participants reported greater symptom burden, underscoring the importance of fatigue-focused, gender-sensitive management strategies.

## 1. Introduction

Rheumatoid arthritis (RA) is a chronic, systemic autoimmune disease that primarily affects peripheral joints and can lead to irreversible joint destruction, disability, and reduced quality of life [1–5]. Beyond musculoskeletal involvement, RA encompasses a broad spectrum of extraarticular complications and psychosocial consequences that significantly impair daily functioning and overall well-being [3–5].

RA disproportionately affects women, with a female-to-male ratio of approximately 3:1, and women consistently report greater pain, fatigue, and disease impact than men [6,7]. Biological, hormonal, and psychosocial mechanisms influence these gender-based disparities. Despite increasing recognition of these differences, the interrelationships among key nonarticular symptoms—particularly fatigue, sleep disturbance, and disease severity—remain poorly understood in the context of gender [7–9].

Fatigue and sleep disturbance are among the most common and debilitating symptoms in RA. Fatigue affects 40–80% of patients, and poor sleep quality—characterized by difficulty initiating and maintaining sleep and nonrestorative rest—is highly prevalent [9–12]. Both symptoms are interrelated: sleep disturbance aggravates fatigue, which in turn exacerbates perceived disease severity and psychological distress [10–13].

Emerging evidence suggests that fatigue may mediate the relationship between poor sleep quality and disease severity. At the same time, gender differences may influence these pathways through hormonal fluctuations, inflammatory activity, or sociocultural stressors. However, few studies have examined whether gender moderates the indirect relationship between sleep quality, fatigue, and disease severity within a unified statistical model.

Therefore, this study aimed to investigate whether fatigue mediates the relationship between sleep quality and disease severity in patients with RA and to determine whether gender moderates these pathways. By integrating a gender-sensitive approach and applying a moderated mediation model, this research seeks to provide a more nuanced understanding of symptom dynamics and inform individualized management strategies in RA.

Research hypotheses:

H1: Fatigue mediates the relationship between sleep quality and disease severity in patients with RA.
H2: The mediating effect of fatigue on the relationship between sleep quality and disease severity is moderated by gender.

## 2. Materials and methods

### 2.1. Study design and participants

This prospective, single-center study was conducted in accordance with the principles of the Declaration of Helsinki and approved by the Institutional Review Board of Ümraniye Training and Research Hospital (approval number: 186-26-05-2022). Inclusion criteria were: (1) confirmed RA diagnosis according to the 2010 American College of Rheumatology/European Alliance of Associations for Rheumatology (EULAR) classification criteria, (2) voluntary participation, and (3) absence of the exclusion criteria. Exclusion criteria included cardiovascular, hepatobiliary, respiratory, renal, or cerebrovascular system dysfunction, and hemoglobin < 11 g/dL. All participants provided written informed consent, and the study was registered in the institutional database.

A total of 68 patients completed the study. The post hoc retrospective sample power was calculated at 0.91 (effect size = 0.35; n = 68), indicating adequate statistical power.

### 2.2. Study instruments

Disease impact was assessed using validated self-report scales alongside demographic and clinical data. Data collection was performed face-to-face by trained research personnel.

A demographic characteristics form was used to collect sociodemographic data (age, gender, marital status, education) and clinical information (disease duration, comorbidities, medications).

The Bristol Rheumatology Fatigue Multidimensional Questionnaire (BRAF–MDQ) was developed to assess the multidimensional impact of fatigue in patients with systemic autoimmune rheumatic diseases and RA. Higher scores indicate greater fatigue [14,15].

Higher Pittsburgh Sleep Quality Index (PSQI) scores reflect poorer sleep quality [16,17].

The Routine Assessment of Patient Index Data-3 (RAPID-3) is a composite index evaluating physical function, pain, and patient global assessment. Higher scores indicate more severe disease severity [18]. The RAPID-3 scale was selected as a validated, multidimensional, and time-efficient patient-reported outcome measure to capture the subjective burden of symptoms. This choice aligns with recent EULAR recommendations emphasizing patient-centered assessment frameworks.

### 2.3. Psychometric properties

The RAPID-3, BRAF–MDQ, and PSQI (Likert items only) demonstrated high reliability. Exploratory factor analysis (EFA) and confirmatory factor analysis (CFA) supported the measurement tools’ structural validity. The factor loadings and fit indices (χ^2^/df < 3) indicated that the PSQI, BRAF–MDQ, and RAPID-3 scales demonstrated adequate psychometric properties in this sample (Table 1) [19].

### 2.4. Statistical analysis

Statistical analyses were conducted using Jamovi software (version 2.6.22) and G*Power (version 3.1.9.7) for post hoc power estimation. Descriptive statistics were calculated for all demographic and clinical variables. Normality was assessed using the Shapiro–Wilk test, and group comparisons were performed with the independent samples t-test, Mann–Whitney U test, chi-square, or Fisher’s exact test, as appropriate.

Pearson correlation analysis was used to examine bivariate associations among sleep quality, fatigue, and disease severity. Multicollinearity was assessed using variance inflation factors (VIFs), and no collinearity issues were detected (all VIFs < 2.0). Logistic regression was performed to examine whether sleep quality, fatigue, and disease severity predict menopausal status among female participants. The mediation and moderation analyses were performed using mediation–moderation modeling, corresponding to the PROCESS macro Models 4 (simple mediation) and 14 (moderated mediation), developed by Hayes (2018). Indirect effects were estimated using 5000 bootstrap samples and 95% bias-corrected confidence intervals. The significance of the indirect effects was further confirmed by the Sobel test (z = 2.97, p = 0.003).

To control for potential biological confounding, C-reactive protein (CRP) was included as a covariate in all models. The inclusion of CRP did not materially alter the mediation or moderated mediation effects. In a subset of participants with available Disease Activity Score 28 (DAS28; n = 22), the correlation patterns were consistent with those of the main analyses, supporting the robustness of the findings. A p < 0.05 was considered statistically significant.

## 3. Results

A total of 68 patients with RA aged 23 to 86 years, with a body mass index (BMI) range of 16.8 to 40.20 and a disease duration of six to 420 months. Among the participants, 55.90% were female, 70.59% were married, 73.53% reported treatment compliance, and 13.24% reported using alternative treatments. Additionally, 22.1% were current smokers, and 8.8% consumed alcohol. As shown in Table 2, there was a statistically significant difference in smoking status between genders (p < 0.05), with male participants smoking more.

As shown in Table 3, female patients exhibited significantly higher disease severity scores (RAPID-3) compared with male patients (5.57 ± 2.23 vs. 4.43 ± 2.12; p = 0.040). Similarly, fatigue severity (BRAF–MDQ) was significantly greater in females (45.05 ± 14.12) than in males (35.83 ± 14.44; p = 0.01). In contrast, sleep quality, as assessed by the PSQI, did not significantly differ between genders (11.58 ± 3.59 vs. 11.33 ± 4.07; p = 0.79). The multidimensional structure of fatigue, as assessed by the BRAF–MDQ, provides valuable insight into the broader symptom experience of individuals with RA. In particular, the cognitive and emotional fatigue subdimensions are closely aligned with the psychological dimensions of fatigue reported in mood-related conditions. Gender differences in the subdimensions of Fatigue in Activities of Daily Living, Cognitive Fatigue, and Emotional Fatigue were not statistically significant (p > 0.05).

A subgroup analysis was conducted among female participants (n = 38) to explore the potential influence of menopausal status on symptom burden. The mean age of menopausal women was 61.60 ± 11.60 years. Although none of the group differences reached statistical significance (p > 0.05), women without menopause showed slightly higher mean scores in sleep disturbance, fatigue, and disease severity (Table 4). These exploratory findings, based on a small subsample, should be interpreted with caution due to limited statistical power. Logistic regression analysis indicated no significant association between menopausal status and sleep quality, fatigue, or disease severity (p > 0.05). Overall, menopausal status may represent a contextual rather than causal factor in symptom variability, warranting confirmation in larger, adequately powered studies.

Correlation analyses demonstrated significant positive associations among sleep quality, disease severity, and fatigue (p < 0.05). Poorer sleep quality (higher PSQI scores) was associated with both greater fatigue severity and higher disease severity. Fatigue severity further showed a strong positive correlation with disease severity (Table 5).

Poorer sleep quality significantly predicted greater fatigue (β = 0.402, p < 0.001), which in turn predicted greater disease severity (β = 0.520, p < 0.001). The indirect effect of sleep quality on disease severity through fatigue was significant (β = 0.209, p = 0.003), while the direct effect was nonsignificant (β = 0.047, p = 0.663), indicating full mediation. Female participants reported higher fatigue levels (β = 0.297, p = 0.005) and slightly higher disease severity scores, although gender did not significantly moderate the mediation pathway (PSQI × gender: β = 0.019, p = 0.856). CRP was included as a covariate to control for inflammatory activity, and its inclusion did not alter the mediation or moderation effects (CRP → BRAF–MDQ → RAPID-3, p = 0.177; CRP → RAPID-3, p = 0.112) (Table 6, Figure). These gendered differences in symptom severity may reflect underlying biological, psychosocial, or behavioral factors that warrant further exploration. Taken together, these findings highlight fatigue as a critical mediator that explains how poor sleep quality and female gender contribute to worsened disease activity in patients with RA. These insights underscore the need to target fatigue management, particularly in female patients, to improve disease outcomes.

## 4. Discussion

The present study examined whether fatigue mediates the relationship between sleep quality and disease severity in patients with RA, and whether this pathway differs by gender. The findings confirmed that poorer sleep quality was associated with greater fatigue, which in turn predicted more severe disease outcomes, supporting a full mediation model. Gender did not moderate this relationship, although women consistently reported higher fatigue and disease severity scores.

These results align with prior studies indicating that fatigue is a central mechanism connecting poor sleep and heightened disease burden in RA [4,10,20]. Fatigue represents a multifactorial symptom influenced by biological, psychological, and behavioral processes. Inflammatory mediators—particularly interleukin-6 (IL-6) and tumor necrosis factor-alpha (TNF-α)—are known to disrupt circadian rhythm and impair hypothalamic–pituitary–adrenal axis regulation, leading to nonrestorative sleep and increased fatigue [20–26]. Chronic inflammation and cytokine imbalance may therefore create a feedback loop that sustains both sleep disturbance and disease activity.

Although women reported greater fatigue and disease severity, the indirect effect of sleep on disease outcomes through fatigue was similar across genders. This suggests that while women experience a higher overall symptom burden, the underlying mechanisms linking these symptoms operate similarly in both sexes. The menopause-related findings in this study should be interpreted as exploratory; hormonal transitions may influence sleep and fatigue, but our cross-sectional design precludes causal interpretation. Future research should further investigate these pathways in larger samples using hormonal and inflammatory biomarkers. As this study relied primarily on patient-reported outcomes, the findings reflect perceived rather than biochemical disease activity. However, CRP levels were considered as a covariate, and the observed relationships remained consistent.

From a clinical perspective, these findings highlight fatigue as a treatable target within the symptom cluster of RA. Routine assessment of fatigue and sleep quality in clinical settings may allow early identification of high-risk patients—particularly women. Nurse-led and multidisciplinary interventions that address fatigue and sleep can improve both physical and psychological well-being. Effective approaches may include sleep hygiene education (consistent sleep–wake schedules, minimizing stimulant use, and optimizing bedtime routines), regular moderate exercise programs that improve energy levels and sleep regulation, and cognitive–behavioral therapy aimed at reducing unhelpful fatigue-related cognitions and promoting adaptive coping strategies.

Integrating these nonpharmacological interventions alongside standard RA management may enhance treatment outcomes and quality of life. Evidence from nurse-led programs, such as COMEDRA, further supports the effectiveness of structured nursing interventions in improving patient-reported outcomes in RA [27].

Kozlowska and Baczyk (2025) reported that approximately 71.1% of female patients with RA had poor sleep quality. Furthermore, significant correlations were found between poor sleep quality and pain intensity, morning stiffness duration, disease activity score (DAS28), and fatigue level. These findings suggest that sleep disturbance in RA may affect not only quality of life but also the severity of disease symptoms and fatigue levels [28]. Similarly, Koc et al. (2025) reported that gender differences in RA have generally been examined and discussed in relation to clinical characteristics and psychosocial outcomes. Fatigue and sleep quality are closely associated with these psychosocial outcomes [29,30].

### Conclusion

Fatigue in RA is a complex, multifactorial symptom shaped by disease-related, behavioral, and demographic factors. This study demonstrated that poor sleep quality contributes to higher fatigue levels and that disease severity serves as a key pathway linking these symptoms. Gender was also found to be an influential factor, with female patients consistently reporting greater fatigue and higher disease severity than their male counterparts.

These findings emphasize the importance of adopting a symptom-cluster approach in the management of RA, addressing fatigue alongside sleep disturbances and disease severity. Implementing gender-sensitive strategies that account for the disproportionate symptom burden experienced by women may enhance clinical decision-making and promote more individualized patient care.

As this study relied entirely on patient-reported outcomes, the results highlight the critical value of incorporating patients’ perspectives in both the evaluation and planning of interventions. Supporting individuals with RA in managing sleep and fatigue may significantly alleviate overall disease burden and improve quality of life.

It is therefore imperative that healthcare providers prioritize fatigue and sleep quality in the routine care of RA patients—especially among women—to achieve more effective, equitable, and patient-centered outcomes.

Future research in larger, longitudinal cohorts should clarify the biological and psychosocial mechanisms underlying these relationships. A multidimensional, gender-informed approach may represent a critical step toward improving outcomes for individuals living with RA. The observed menopausal patterns were exploratory and should not be generalized without further replication in larger samples.

To our knowledge, this is among the first international studies—and the first from Turkiye—to apply a gender-based moderated mediation framework to examine the interrelationships among sleep quality, fatigue, and disease severity in RA. Unlike previous research limited to bivariate or regression analyses, this study provides a novel, multidimensional perspective that highlights fatigue as the central mechanism linking sleep and disease severity. This originality not only advances conceptual understanding but also offers clinically actionable insights for gender-sensitive management strategies.

This study has several limitations that should be acknowledged. First, although the sample size (n = 68) was sufficient for statistical analysis, it may limit the generalizability of the findings to the broader RA population. Second, the cross-sectional design does not allow for causal or temporal inferences regarding the relationships between sleep quality, fatigue, and disease severity. Third, several potential confounding factors—such as psychological comorbidities (e.g., depression, anxiety), medication use, menopausal status, and socioeconomic variables—were not systematically controlled and may have influenced symptom expression. Although depression and anxiety were not assessed as independent variables, the multidimensional structure of the BRAF–MDQ, which includes cognitive and emotional components, indirectly captured certain aspects of psychological burden.

Importantly, the exclusion of potential confounders such as disease-modifying antirheumatic drug regimen, pain intensity, physical activity level, and inflammatory markers (e.g., CRP, erythrocyte sedimentation rate) may have influenced both the observed associations and the model’s predictive accuracy. These variables are known to interact with sleep quality, fatigue, and disease perception in RA. Their omission might have led to over- or underestimation of the indirect effects detected in the mediation model. Consequently, the current findings should be interpreted with caution, as they primarily reflect self-reported symptom interrelations rather than comprehensive biopsychosocial mechanisms. Future studies should include these factors in multivariate or longitudinal models to better isolate the unique contributions of sleep and fatigue to disease severity.

Future research should include larger, more diverse samples and adopt longitudinal or interventional designs to confirm the current findings and clarify the directionality of associations between sleep disturbances, fatigue, and disease severity—particularly in relation to gender differences. Given the well-established impact of depression and anxiety on fatigue and sleep quality in RA, assessing these factors explicitly in future studies may yield more comprehensive insights. Lastly, the exclusive use of self-reported measures may introduce recall or reporting bias.

## Figures and Tables

**Figure:**
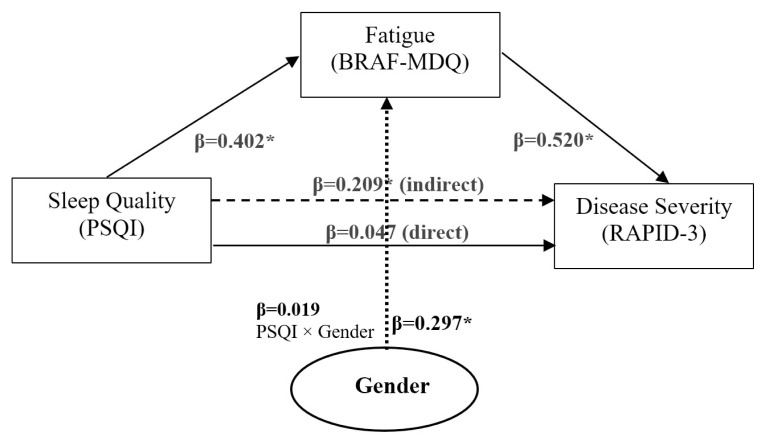
Moderated mediation model examining the indirect effect of sleep quality (PSQI) on disease severity (RAPID-3) through fatigue (BRAF–MDQ).

**Table 1:** Reliability and validity indices of the study instruments (RAPID-3, BRAF–MDQ, PSQI).

	aCronbach’s α	aMcDonald’s ω	bKMO	bBartlett’s test of sphericity (χ^2^)	cχ^2^/df
RAPID-3	0.773	0.925	0.814	509*	2.86
BRAF–MDQ	0.911	0.937	0.827	833*	3.24
PSQI	0.815	0.812	0.669	545*	2.98

α: Cronbach’s alpha, ω: McDonald’s omega, KMO: Kaiser–Meyer–Olkin measure of sampling adequacy, χ^2^: chi-square;

a reliability analysis,

b EFA,

c CFA.

* statistically significant (p < 0.05)

**Table 2:** Descriptive statistics and analysis of participants by gender.

Variables	Female n = 38 (55.90%)	Male n = 30 (44.10%)	p-value	Total
Age (mean ± SD)	53.79 ± 15.33	51.90 ± 14.72	a0.61	52.96 ± 14.98
BMI (mean ± SD)	28.18 ± 5.06	27.27 ± 5.91	a0.50	27.78 ± 5.43
Durations (mean ± SD)	93.05 ± 76.49	76.73 ± 87.82	b0.08	85.85 ± 81.46
Marital status n (%)			c0.93	
Married	27 (71.05%)	21 (70.00%)		48 (70.59%)
Single	11 (28.95%)	9 (30.00%)		20 (29.41%)
Smoking n (%)			c0.007*	
Smoker	6 (15.79%)	9 (30.00%)		15 (22.06%)
Nonsmoker	27 (71.05%)	10 (33.33%)		37 (54.41%)
Former smoker	5 (13.16%)	11 (36.67%)		16 (23.53%)
Alcohol consumption n (%)			c0.93	
Drinker	3 (7.89%)	3 (10%)		6 (8.82%)
Nondrinker	29 (76.32%)	23 (76.67%)		52 (76.47%)
Former drinker	6 (15.79%)	4 (13.33%)		10 (14.71%)
Treatment compliance n (%)	27 (71.05%)	23 (76.67%)	c0.60	50 (73.53%)
Alternative treatment n (%)	3 (7.89%)	6 (20%)	d0.17	9 (13.24%)

a t-test

b Mann–Whitney U Test

c chi-square

d Fisher’s exact test;

* statistically significant (p < 0.05)

**Table 3:** Descriptive statistics for disease severity, sleep quality, and fatigue.

Variables	Female	Male	a p-value	Total
Skill (mean ± SD)	3.33 ± 2.59	2.27 ± 1.95	0.07	2.86 ± 2.37
Pain (mean ± SD)	6.47 ± 3.31	5.6 ± 3.43	0.29	6.09 ± 3.37
Health status (mean ± SD)	5.91 ± 2.94	4.57 ± 2.41	0.04*	5.32 ± 2.78
Disease Severity: RAPID-3 (mean ± SD)	5.57 ± 2.23	4.43 ± 2.12	0.04*	5.07 ± 2.24
Sleep quality: PSQI (mean ± SD)	11.58 ± 3.59	11.33 ± 4.07	0.79	11.47 ± 3.78
Physical fatigue (mean ± SD)	17.30 ± 4.46	13.86 ± 6.35	0.01*	15.79 ± 5.6
Fatigue in Activities of Daily Living (mean ± SD)	13.73 ± 5.72	11.4 ± 5.21	0.09	12.69 ± 5.58
Cognitive fatigue (mean ± SD)	6.51 ± 3.59	5.53 ± 3.46	0.26	6.07 ± 3.54
Emotional fatigue (mean ± SD)	7.16 ± 2.92	5.73 ± 3.69	0.08	6.52 ± 3.34
Fatigue total: BRAF–MDQ (mean ± SD)	45.05 ± 14.12	35.83 ± 14.44	0.01*	40.99 ± 14.88

a t test

* statistically significant (p < 0.05)

**Table 4:** Comparison of sleep quality, fatigue, and disease activity by menopausal status (exploratory analysis).

	Menopause (+)	Menopause (−)	p	Total
PSQI (n = 38)	11.20 ± 3.91 (n = 25)	12.31 ± 2.9 (n = 13)	0.308	11.58 ± 3.59
RAPID-3 (n = 38)	5.42 ± 2.53 (n = 25)	5.87 ± 1.53 (n = 13)	0.963	5.57 ± 2.23
BRAF–MDQ (n = 38)	44.84 ± 16.38 (n = 25)	45.46 ± 8.8 (n = 13)	0.681	45.05 ± 14.12
DAS 28 (n = 11)	4.22 ± 0.65 (n = 8)	4.48 ± 0.58 (n = 3)	0.781	4.29 ± 0.62

**Table 5:** Correlation between sleep quality, fatigue, and disease activity.

r	PSQI	RAPID-3	BRAF–MDQ
PSQI (n = 68)	—		
RAPID-3 (n = 68)	0.264*	—	
BRAF–MDQ (n = 68)	0.411*	0.565*	—
DAS 28 (n = 22)	0.439*	0.437*	0.462*

r: Pearson correlation coefficients

* statistically significant (p < 0.05)

**Table 6:** Moderated mediation analysis of the relationship between sleep quality (PSQI), fatigue (BRAF–MDQ), and disease severity (RAPID-3), moderated by gender.

Type/effect	Estimate	95% CI	β	p
**Indirect**				
aPSQI ⇒ BRAF–MDQ	0.124	0.042–0.206	0.209	0.003*
aGender ⇒ BRAF–MDQ	0.692	0.131–1253	0.154	0.016*
aGender: PSQI ⇒ BRAF–MDQ	0.012	−0.115–0.138	0.010	0.856
**Component**				
bPSQI	1.580	0.774–2387	0.402	<0.001*
aBRAF–MDQ	0.078	0.045–0.112	0.520	<0.001*
bGender	8.833	2.736–14.931	0.297	0.005*
bGender: PSQI	0.149	−1.463–1.762	0.019	0.856
**Direct**				
aPSQI	0.028	−0.097–0.152	0.047	0.663
aGender	0.415	−0.487–1317	0.093	0.367
aGender: PSQI	−0.210	−0.435–0.016	−0.177	0.069
**Total**				
aPSQI	0.151	0.021–0.282	0.256	0.023*
aGender	1.107	0.122–2092	0.247	0.028*
aGender: PSQI	−0.198	−0.458–0.063	−0.167	0.137

a Gender (female);

CI = confidence interval; PSQI = Pittsburgh Sleep Quality Index; BRAF–MDQ = Bristol Rheumatoid Arthritis Fatigue; RAPID-3 = Routine Assessment of Patient Index Data3.

a dependent variable: RAPID-3;

b dependent variable: BRAF–MDQ

* statistically significant (p < 0.05)
